# Rare Germline Genetic Variants and the Risks of Epithelial Ovarian Cancer

**DOI:** 10.3390/cancers12103046

**Published:** 2020-10-19

**Authors:** Marina Pavanello, Isaac HY Chan, Amir Ariff, Paul DP Pharoah, Simon A. Gayther, Susan J. Ramus

**Affiliations:** 1 School of Women’s and Children’s Health, Faculty of Medicine, University of New South Wales, Sydney 2052, Australia; m.pavanello@student.unsw.edu.au (M.P.); isaac.chan@unsw.edu.au (I.H.C.); amir.ariff@unsw.edu.au (A.A.); 2 Adult Cancer Program, Lowy Cancer Research Centre, University of New South Wales, Sydney 2052, Australia; 3 Strangeways Research Laboratory, University of Cambridge, Cambridge CB1 8RN, UK; pp10001@medschl.cam.ac.uk; 4 Center for Cancer Prevention and Translational Genomics, Cedars Sinai Medical Center, Los Angeles, CA 90048, USA; simon.gayther@cshs.org; 5 Applied Genomics, Computation and Translational Core, Cedars Sinai Medical Center, Los Angeles, CA 90048, USA

**Keywords:** ovarian cancer risk, rare germline variants, susceptibility genes

## Abstract

**Simple Summary:**

Several genes have been confirmed as risk genes for epithelial ovarian cancer (EOC). There are five main types of EOC, with different molecular changes and clinical characteristics, suggesting they should be considered different diseases. This review summarises the contribution of rare inherited mutations to EOC susceptibility, focussing on the frequency in each EOC type. Susceptibility genes can have a major clinical impact, reducing ovarian cancer incidence by screening of family members to detect women at higher risk than the general population. They can also lead to the development of new targeted treatments.

**Abstract:**

A family history of ovarian or breast cancer is the strongest risk factor for epithelial ovarian cancer (EOC). Germline deleterious variants in the *BRCA1* and *BRCA2* genes confer EOC risks by age 80, of 44% and 17% respectively. The mismatch repair genes, particularly *MSH2* and *MSH6*, are also EOC susceptibility genes. Several other DNA repair genes, *BRIP1*, *RAD51C*, *RAD51D*, and *PALB2*, have been identified as moderate risk EOC genes. EOC has five main histotypes; high-grade serous (HGS), low-grade serous (LGS), clear cell (CCC), endometrioid (END), and mucinous (MUC). This review examines the current understanding of the contribution of rare genetic variants to EOC, focussing on providing frequency data for each histotype. We provide an overview of frequency and risk for pathogenic variants in the known susceptibility genes as well as other proposed genes. We also describe the progress to-date to understand the role of missense variants and the different breast and ovarian cancer risks for each gene. Identification of susceptibility genes have clinical impact by reducing disease-associated mortality through improving risk prediction, with the possibility of prevention strategies, and developing new targeted treatments and these clinical implications are also discussed.

## 1. Introduction

Epithelial ovarian cancer (EOC) is the seventh most common cancer in women worldwide, with over 295,000 incident cases each year, and is the leading cause of mortality relating to gynaecological malignancies, with 184,000 deaths each year [1]. Typically, EOCs are stratified into five main histological subtypes: High grade serous, which account for up to 70% of all EOC cases, endometrioid (~10%), clear cell (~10%), mucinous (~3%), and low-grade serous carcinomas (<5%) [2]. The five different histotypes have different risk factors and molecular characteristics. The inherited genetics of each histotype is also likely to be different.

The prognosis of ovarian cancer is poor, with a five-year survival rate of just 47% [3]. Ovarian cancer is difficult to diagnose early in its disease course, and 80% of cases are diagnosed after extensive metastasis at stage III or IV, which carry a five-year survival rate of 41% and 20% respectively [3]. However, outcomes are good if ovarian cancer is detected early, with an 89% five-year survival in stage I cancers [3]. Screening for ovarian cancer is of limited utility and no studies investigating ovarian cancer screening have shown a significant impact of screening on mortality [4,5]. As a result, population-based screening for ovarian cancer is not recommended in the guidelines of any major society. As the prognosis of EOC is related to its stage at diagnosis, the ability to use genetic information to predict EOC risk and intervene before disease development would reduce overall mortality and morbidity in ovarian cancer [6].

A family history of EOC confers an increase in relative risk (RR) of ovarian cancer of 2.96. The known EOC risk genes explain less than half of the excess familial risk for EOC, suggesting that there are still undiscovered ovarian cancer predisposing genes to be found [7,8]. The known ovarian cancer predisposing genes are from two different DNA repair pathways. The homologous recombination (HR) DNA repair pathway and the mismatch repair (MMR) DNA pathway. The majority of the genes are part of the HR DNA repair pathway. *BRCA1* and *BRCA2* have been confirmed as highly penetrant predisposition genes for EOC [9]. Several other genes—*BRIP1*, *RAD51C*, *RAD51D*, and *PALB2*, recently described as moderate risk genes, are also part of this pathway [10,11,12]. The mismatch repair genes *MLH1*, *MSH2*, *MSH6*, and *PSM2* are also confirmed moderate risk EOC susceptibility genes [13]. The frequency of disease-associated variants in these genes is different for the different EOC histotypes. Thirty-seven common variants have been identified for ovarian cancer, using genome-wide association studies (GWAS) [14,15]. The ability to derive accurate risk estimates for ovarian cancer from genetic information in asymptomatic women has significant implications for ovarian cancer management.

This review aims to summarise the progress to date in efforts to understand the contribution of rare genetic variants to ovarian cancer and histotype specific differences, as well as the clinical implications of these discoveries. In the first part of this review, we describe germline protein truncating variants (insertion/deletions or nonsense) in confirmed EOC susceptibility genes, followed by an overview of the role of missense variants in these genes. Lastly, we described germline protein truncating variants in other proposed genes that have not been validated as EOC susceptibility genes.

## 2. Protein Truncating Variants in Confirmed Susceptibility Genes

### 2.1. BRCA1 and BRCA2

These two major breast/ovarian cancer susceptibility genes encode proteins that work in DNA replication pathways to avoid double-strand DNA damage. Deleterious protein truncating variants include small insertions and deletions and single base changes, as well as large genomic alterations, which account for approximately 10% of *BRCA1* variants [16]. Several deleterious missense variants have also been described.

The prevalence and penetrance of deleterious variants in *BRCA1* and *BRCA2* has been extensively studied in ovarian cancer patients. The case ascertainment, either from families with a history of breast or ovarian cancer or from the general ovarian cancer population, has a major impact on the results. The presence of founder mutations in the population also affects the prevalence and has important implications for clinical testing. The presence of founder mutations can allow for cheap and rapid testing and the potential for population screening. In the Ashkenazi Jewish population, there are three *BRCA* founder mutations (185delAG and 5382insC in *BRCA1* and 6174delT in *BRCA2*) that accounts for almost all mutations present in this population [17]. In the Icelandic population, there is a single founder mutation, 999del5, in the *BRCA2* gene. Most of the breast and ovarian cancer cases in Icelandic families have this *BRCA2* founder mutation, while mutations in *BRCA1* are very rare [18]. Founder mutations in the *BRCA* genes have also been reported in many other populations, including Russians [19,20], Polish [21], Norwegian [22], Finnish [23], Japanese [24], and Chinese [25]. While some mutations are restricted to isolated regions or certain populations, others are common across different countries, such as *BRCA1* 5382insC mutation that is common in many European populations [17].

Early studies of cases selected for a family history of breast or ovarian cancer found that 24–76% had deleterious variants in *BRCA1* and 1–17% had deleterious variants in *BRCA2* [17]. Recent studies using clinical testing laboratory data have reported the prevalence of *BRCA1/2* pathogenic variants in 5020 and 7489 cases from the USA [26,27], 4409 cases from France [28], and 3230 cases from a meta-analysis of 48 multi-gene panel testing-based studies [29]. In these studies, the frequency of germline pathogenic variants in *BRCA1* in ovarian cancer cases were 5.1%, 3.6%, 3.7%, and 8.6%, respectively, and 3.9%, 3.3%, 4.0%, and 4.5% in *BRCA2* [26,27,28,29].

Testing of unselected ovarian cancer cases in non-Ashkenazi Jewish populations shows that the frequency of deleterious *BRCA1* and *BRCA2* variants ranged from 3–10% and 0.6–6%, respectively [17]. Recent population-based studies from Table 1 found that 3.8–11.1% of cases have *BRCA1* deleterious variants and 4.3–6.4% had *BRCA2* deleterious variants.

In a large prospective study of *BRCA* carriers ascertained through family clinics, the cumulative risk, by age 80, of ovarian cancer in women with pathogenic variants in *BRCA1* and *BRCA2* was estimated to be 44% (95% confidence interval (CI), 36–53%) and 17% (95% CI, 11–25%), respectively [9]. The histotypes of ovarian cancer diagnosed in the study were not described [9]. Breast cancer was also assessed and the cumulative risk at the age of 80 years was estimated to be 72% (95% CI, 65–79%) for *BRCA1* carriers and 69% (95% CI, 61–77%) for *BRCA2* [9]. Variants in these *BRCA* genes are also associated with increased risk of pancreatic cancer [30] and high-grade prostate cancer [31].

Four studies, with cases unselected for age or family history, provided information on histotype (Table 1). Due to the small numbers of the much rarer histotypes (END, CCC, LGS, and MUC), the frequencies of germline pathogenic variants varied significantly. In an effort to estimate the frequency in these rarer histotypes, we have combined the results across studies. This showed that the frequency of deleterious variants in *BRCA1* were approximately 7.8% in HGS, 3% in END, 3.6% in CCC, 3.7% in LGS, and <1% in MUC [32,33,34,35]. In *BRCA2*, deleterious variants were seen in approximately 5.9% of HGS cases, 2.9% of END, 0.9% of CCC, 2.0% of LGS, and <1% of MUC [33,34,35] (Table 1). According to these data, both genes have a higher frequency of variants in the HGS histotype compared to the non-HGS histotypes, and a much lower frequency in mucinous cases. Available cumulative risks from these retrospective studies are comparable to the prospective study previously mentioned [9] (Table 1).

Ovarian cancer patients with deleterious variants in *BRCA1* and *BRCA2* are characterized by genomic instability within their tumours. They have a better response to platinum-based chemotherapies, resulting in improved five-year overall survival. Five-year overall survival for non-carriers was reported to be 36% (95% CI 34–38) compared to 44% (95% CI 40–48) for *BRCA1*-carriers and 52% (95% CI 46–58) for *BRCA2*-carriers [36]. However, this survival advantage was not present when follow-up was extended to 10 years [37,38].

Identifying pathogenic variants associated with increased risk of ovarian cancer has major clinical implications. Due to the magnitude of ovarian cancer risk associated with variants in *BRCA1* and *BRCA2*, risk-reducing salpingo-oophorectomy (RRSO) is currently recommended as a prevention strategy for *BRCA1* carriers by age 35 to 40 years, once the woman’s childbearing is complete, and for *BRCA2* carriers by age 40 to 45 [39].

Furthermore, understanding the role of *BRCA1/2* variants in ovarian cancer has allowed for the development of targeted therapies, namely PARP (poly[adenosine diphosphate–ribose] polymerase) inhibitors, which improve progression-free survival in selected women with ovarian cancer. Approximately 50% of HGS cases present defects in DNA repair mechanisms due to pathogenic variants in the homologous recombination deficiency (HRD) genes, such as *BRCA1/2*, or due to functional inactivation through methylation [40]. PARPi have shown highly efficacious activity particularly in women with platinum-sensitive disease carrying *BRCA1/2* variants, or women with other homologous recombination deficiencies [41,42,43]. Improved activity has also been seen in women with recurrent disease regardless of their *BRCA* status [44]. As *BRCA1/2* status affects clinical management of affected women, current guidelines recommend *BRCA1/2* testing in all non-mucinous ovarian cancer cases [39].

### 2.2. BRIP1

The protein encoded by *BRIP1* is part of the Fanconi anaemia group (FANCJ) and is involved in the repair of DNA double-strand breaks by homologous recombination. *BRIP1* was described as a candidate risk gene by Walsh et al. that identified germline loss-of-function variants in 1% of ovarian cancer cases not selected for age or family history [34]. Pathogenic germline variants in *BRIP1* are the most common mutation found in ovarian cancer after *BRCA1/2* with a frequency of approximately 1% of the EOC cases [11].

The contribution of germline protein truncating variants in *BRIP1* have not yet been assessed in prospective studies. Ramus et al. described a relative risk associated with protein truncating variants in *BRIP1* of 11.2 (95% CI 3.2–34.1), with an estimated cumulative risk of 5.8% (95% CI 3.6–9.1%) by the age of 80 years [11] (Table 2). The association between protein truncating variants in *BRIP1* and risk of ovarian cancer has been confirmed in other analyses (associated risks ranged from 2.6 to 6.4 [27,28,29,35,45]) (Table 2 and Table 3). The frequency of protein truncating variants in population-based studies, separated by ovarian cancer histotypes are given in Table 2, and the frequency in the other retrospective studies, mostly family studies or with women referred to clinical testing, are given in Table 3. Some of the studies in Table 3 have included predicted deleterious missense changes that cannot be separated from the totals, and these are indicated.

Three studies have reported the frequency of protein truncating variants in *BRIP1* by ovarian cancer histotype. Combining these data show that approximately 1.2% of HGS and END cases and 0.8% of LGS cases had protein truncating variants in *BRIP1* [11,34,35] (Table 2). No variants were observed in CCC and MUC, but the total number of cases examined were low. Additional studies are needed to further assess the contribution of germline protein truncating variants in *BRIP1* in the ovarian cancer histotypes.

Available data for *BRIP1* consistently have shown an increased risk of ovarian cancer, with cumulative risk estimated to be approximately 6% by age of 80, and the National Comprehensive Cancer Network, USA, now recommends RRSO for women, starting from ages 45 to 50 [39].

### 2.3. RAD51C and RAD51D

*RAD51C* and *RAD51D* are homologous recombination genes, which encode proteins that interact with *BRCA1/2* and participate in the DNA repair process. Ovarian cancer risk attributed to protein truncating variants in *RAD51C* and *RAD51D* was first described by Meindl et al. in 2010 and Loveday et al. in 2011 [46,47].

Song et al. and Norquist et al. provided the frequency of protein truncating variants in the *RAD51C/D* genes in population-based studies by ovarian cancer histotypes [35,48] (Table 2). Combining their data, approximately 0.4–0.5% of HGS, END and CCC cases and 0.2% of LGS had protein truncating variants in *RAD51C*. For *RAD51D*, 0.5% of HGS cases and 0.9% of END cases had protein truncating variants. These variants were not detected in CCC, LGS, and MUC cases, although so far, the number of cases of these histotypes in the datasets are low. The frequency of deleterious variants in family-based studies that combined all EOC cases are given in Table 3. The estimated risk associated with *RAD51C/D* described in the two population-studies were OR 5.2 (95% CI 1.1–24) and 3.4 (95% CI 1.5–7.6) for *RAD51C* and 12.0 (95% CI 1.5–90) and 10.9 (95% CI 4.6–26) for *RAD51D* (Table 2) [35,48]. Three additional case-control studies which included only individuals referred to clinical testing estimated a risk of OR 4.9 (95%CI 3.0–8.0), OR 14.6 (95% CI 5.3 –29.5), and SRR 5.12 (95% CI 3.7–6.9) for *RAD51C*. For *RAD51D*, associated risks were OR 4.78 (95% CI 2.1–10.7), OR 11.8 (95% CI 1.1–40), and SRR 6.34 (95% CI 3.1–11.3) (Table 3) [26,27,28].

More recently, Yang et al. performed a study of 125 families with pathogenic variants in *RAD51C* and 60 families with pathogenic variants in *RAD51D* and confirmed an increased risk of ovarian cancer associated with pathogenic variants in both genes (*RAD51C*, RR 7.55, 95% CI 5.6–10.2 and *RAD51D*, RR 7.6, 95% CI 5.6–10.3) [10]. The cumulative risk of having ovarian cancer by age 80 was estimated to be 11% (95% CI 6–21%) for *RAD51C* and 13% (95% CI 7–23%) for *RAD51D* based on their segregation analysis [10] (Table 3). *RAD51C/D* were also shown to be associated with breast cancer with cumulative risk of 21% (95% CI 15–29%) and 20% (95% CI 14–28%) for these genes by the age of 80 years, respectively [10].

There is no consensus about the EOC risk threshold for surgical prevention, although the acceptability of RRSO for women with a lifetime risk greater than 10% is well-established. It has been recently suggested that this threshold should be lower and was demonstrated that prophylactic surgery is cost-effective for women at lifetime risk of 5% [49,50,51]. Considering the current cumulative risk estimates for *RAD51C* and *RAD51D*, women carrying pathogenic variants in these genes could be offered preventive surgery starting from ages 45 to 50, once childbearing is complete.

### 2.4. PALB2

*PALB2* is a confirmed breast cancer susceptibility gene with a cumulative risk estimated to be 44% by 80 years [52], and therefore clinical testing for germline pathogenic variants in this gene is part of breast cancer standard of care. *PALB2* is needed to recruit *BRCA2* in the HR DNA repair pathway [53].

Initial analysis of 3227 EOC cases and 3444 matched-controls suggested that larger numbers of samples were required to determine if *PALB2* was an ovarian cancer susceptibility gene (p 0.08) [11]. Evidence of risk association was observed in case-control analysis of 1915 EOC cases compared to publicly available controls with the OR estimated to be 4.4 (95% CI 2.1–9.1) [35]. Recently, *PALB2* has been confirmed as a risk gene by targeted sequencing in 5123 HGS cases and 5202 controls, WES data from 829 cases and 913 controls, and genotyping data from an independent set of ~14,000 EOC cases and 29,000 controls [54]. The odds ratio was estimated to be 3.01 (95% CI 1.59–5.68) [54] (Table 2).

Histotype data are only available from the first two studies, and combined show that approximately 0.4% of HGS cases had protein truncating variants in *PALB2* (Table 2). A lower frequency was found for LGS (0.2%) and no variants were found in END and MUC. In contrast CCC cases had a frequency of 2.4%. The total number of non-HGS cases examined to date, particularly for CCC, is very low, thus larger numbers are needed to establish the true frequency of *PALB2* pathogenic variants in the rarer histotypes.

An increased risk of ovarian cancer has recently been confirmed in a study of 976 individuals with protein truncating variants in *PALB2* from 524 families, where complex segregation analysis adjusted for ascertainment was performed. The relative risk was estimated to be 2.91 (95% CI 1.4–6.0) [12] (Table 3). Cumulative risks were estimated to be approximately 5% (95% CI 2–10%) by the age of 80 years in the family-based study [12] and 3.2% (95% CI 1.8–5.7%) by the same age in the case-control study previously described [54]. Discussions in the clinical community on whether or not these women should be eligible for prophylactic surgery are still ongoing.

Yang et al. also showed that protein truncating variants in *PALB2* were associated with increased risk of female and male breast cancer (RR 7.18 95% CI 5.8–8.8 and 7.34 95% CI 1.2–42.8, respectively) and pancreatic cancer (RR 2.37 95% CI 1.2–4.5) [12].

### 2.5. Mismatch Repair (MMR) Genes

Germline protein truncating and known deleterious missense variants in four MMR genes are associated with Lynch syndrome, which is an inherited disorder that increases the risk of many types of cancers including colon, endometrial, ovarian, pancreatic, small-bowel, and ureteric. In ovarian cancer cases from Lynch syndrome families, deleterious variants in the MMR genes were mostly seen in END and CCC cases and were more prevalent in the *MSH2* and *MSH6* genes [56]. This histotype-specific characteristic has been confirmed by population-based cohorts of EOC [33,34].

Germline deleterious MMR gene variants in ovarian cancer cases appear to be very rare, which may partly be because they are associated with the rare histotypes of disease [33]. Three studies have described MMR gene mutation frequencies by ovarian cancer histotypes [33,34,35] (Table 4).

We have combined the data across studies to provide an estimate of the frequency in these rarer histotypes (Table 4). Deleterious variants in these genes were more frequent in END and CCC cases than HGS. No variants were reported in MUC or LGS cases, although some studies combined HGS and LGS cases. Variants were more frequently found in *MSH6* than the other genes, with CCC and END cases both having greater than 1% frequency.

Five other case-control studies have investigated MMR frequencies between 2000 and 7500 EOC cases, four of which included data from commercial laboratories where clinical grade testing was performed in women with hereditary cancer risk [26,27,28,57], while another was a meta-analysis [29]. These studies have estimated the OR for mutation carriers of these genes. For *MLH1* carriers the OR was 3 (95% CI, 1.47–6.59) [26], for *MSH2* carriers, OR data from different studies ranged from 2 to 14 [26,27,28,29], and for *MSH6* carriers, ranged from 2 to 9 [26,27,28,29,57]. For the MMR genes combined, the OR was estimated to be 2.3 (95% CI, 0.83–8.2) with a cumulative risk of ovarian cancer by age 80 years of 3.7% (95% CI, 1.4–13%) in cases from a population-based study [33].

A Lynch syndrome prospective study had estimated the cumulative risk of ovarian cancer as a unique disease to be 10% (95% CI 4.8–15.4%) for *MLH1* carriers by the age of 75 years, 17% (95% CI 5.7–28.0%) for *MSH2* carriers, and 13% (95% CI 0.1–31.2%) for *MSH6* carriers [13]. The risk in the different EOC histotypes was not reported [13], although it is known that they are mostly seen in END and CCC cases.

The cumulative risk of endometrial cancer by the age of 75 years was estimated to be 43% (95% CI 33.1–52.3%), 57% (95% CI 41.8–71.6%), and 46% (95% CI 27.3–65.0%) for *MLH1*, *MSH2*, and *MSH6* carriers, respectively [13]. Due to the increased risk of both ovarian and endometrial cancer in women with Lynch syndrome, discussion about RRSO with hysterectomy is recommended. MMR carriers from these Lynch syndrome families were also reported to have an increased risk of colorectal, upper gastrointestinal, urinary tract, prostate, and brain cancers [13].

## 3. Missense Variants in Confirmed Susceptibility Genes

Protein truncating variants have been classified as deleterious, however a relatively large number of missense variants, including some rare predicted pathogenic missense variants have also been identified in these genes. Determining if any of these missense variants are also pathogenic is important for patient management and risk prediction in families.

### 3.1. BRCA1/2 and MMR Genes

Over many years, due to efforts of commercial labs, independent research groups, and large consortia, many missense variants of unknown significance (VUS) or unclassified variants (UV) have been reclassified as either pathogenic or benign. However, there are still many variants that remain unclassified. Large-scale consortia efforts, such as the Evidence-based Network for the Interpretation of Germline Mutant Alleles (ENIGMA) are in the process of investigating and classifying these variants in the *BRCA1/2* genes and the interpretation of the results of genetic testing for reporting and counselling [58].

To date, the National Institutes of Health (NIH) repository, ClinVar, has reported approximately 7000 pathogenic or likely pathogenic variants and 7400 VUS in *BRCA1/2* genes (mostly missense changes, but also in-frame deletions and insertions, and intronic and exonic variants that may affect splicing efficiency) [59]. The same clinical challenge occurs in patients with variants in the MMR genes, approximately 30% of all variants are classified as VUS [60].

Individual studies may include analysis of missense variants using bioinformatic tools such as SIFT [61], PolyPhen-2 [62], and Provean [63] to predict the functional effect of variants. Association between predicted deleterious rare missense variants and ovarian cancer risk can be assessed using burden tests, such as the rare admixture maximum likelihood test (RAML), that accounts for differences in risk of each associated variant [64]. Song et al. have performed this type of analysis in 131 predicted deleterious missense variants in *BRCA1/2* and the MMR genes (*MLH1*, *MSH2*, *MSH6*, and *PMS2*) and found little evidence for ovarian cancer risk association between these variants in any of the six genes [33].

### 3.2. BRIP1, RAD51C/D and PALB2

Missense variants in the moderate-risk genes recently confirmed as susceptibility genes in ovarian cancer need further investigation to determine if any are likely to be pathogenic. Some studies have included predicted deleterious missense variants in estimating mutation frequencies.

Ramus et al. used SIFT, PolyPhen-2, and Provean scores to predict if uncommon (minor allele frequency (MAF) < 1%) and rare (MAF < 0.1%) missense variants were potentially deleterious and identified 35 for *BRIP1* and 26 in *PALB2.* They performed the RAML burden test and found an increased risk association for uncommon and rare missense variants in *BRIP1*, but not for *PALB2* [11,54].

In *RAD51C/D*, Song et al. performed the RAML test for 28 rare predicted deleterious missense variants (12 in *RAD51C* and 16 in *RAD51D*) [48]. Evidence for an association of rare predicted deleterious missense variants with an increased risk of ovarian cancer was observed in both genes [48] (Table 5).

The data from the RAML burden test suggest that some of the predicted deleterious missense variants in *BRIP1*, *RAD51C*, and *RAD51D* likely increase disease risk, but it does not indicate how many or which ones. There is no evidence for specific missense variants.

## 4. Deleterious Variants in Other Proposed Susceptibility Genes

### 4.1. FANCM

*FANCM* is part of the Fanconi anemia group together with *BRCA2*, *BRIP1* and *PALB2* (also known as *FANCD1*, *FANCJ*, and *FANCN*). A case-control study was performed by Dicks et al. where *FANCM* targeted sequencing was performed in 3107 HGS cases, 1491 cases of other histotypes, and 3368 unaffected matched controls [66]. A significantly higher frequency of protein truncating variants was found in the HGS cases compared to the controls (*p* 0.008) and no evidence of association was observed with other histotypes (*p* 0.82) [66]. The relative risk for HGS was estimated to be 2.5 (95% CI 1.3–5) with lifetime average risk of 3.8% (80% CI 2.2–4.5%) [66]. Lifetime risk was increased when known ovarian cancer risk factors such as common risk alleles and lifestyle factors were taken into account (4.6% (80% CI 3.1–7.0%) and 5.2% (80% CI 3.4–7.8%), respectively) [66]. Additionally, the RAML test was performed for 243 uncommon predicted deleterious missense variants (effect given by at least two of the three function prediction programs Polyphen-2, Provean, and SIFT), but there was no difference in the frequency of these variants in cases compared to controls [66].

Although this study had shown evidence of association between germline protein truncating variants in *FANCM* and moderate increase in risk of HGS ovarian cancer, larger and prospective studies are still needed to confirm this.

### 4.2. ATM

*ATM* is a candidate ovarian cancer susceptibility gene due to its role in breast [67] and pancreatic cancer [68]. Germline heterozygous pathogenic *ATM* variants are associated with fivefold higher risk of breast cancer by the age of 50 years [67] and some rare variants have been shown to have penetrance as high as the *BRCA2* gene [69].

In ovarian cancer, case-control studies mainly enriched for a family history of breast or ovarian cancer, have suggested that pathogenic or likely pathogenic variants in *ATM* might be associated with moderate increased risk (OR 1.69, 95% CI 1.2–2.4 [26]; Standardized risk ratio (SRR) 2.25, 95% CI 1.7–3 [27]; OR 1.97, 95% CI 1.3–3 [29]; OR 2.4, 95% CI 1.2–4.7 [35]; OR 2.85, 95% CI 1.3–6.3 [57]), however the cumulative lifetime risk has been estimated to be lower than 3%.

### 4.3. BARD1 and NBN

The *BARD1* and *NBN* genes were included in breast/ovarian cancer genetic panel tests, due to their breast cancer risk, despite the ovarian cancer risk for deleterious variants in these genes being unknown. The *BARD1* encoded protein interacts closely with the *BRCA1* protein due to sharing the N-terminal RING finger and the BRCA1 C-terminal domains. Interaction between these genes affects double-strand break repair and apoptosis suggesting that this protein may play an important role in *BRCA1* tumour suppression [70]. Consequently, *BARD1* was considered a potential candidate susceptibility gene for ovarian cancer. However, Ramus et al. found no significant differences in *BARD1* deleterious variants frequency in a cohort of ~3200 ovarian cancer cases compared with ~3400 matched-controls (*p* 0.39) [11]. Several additional case-control studies confirmed that no evidence of association was observed between ovarian cancer risk and pathogenic variants in *BARD1* (OR 0.59, 95% CI 0.21–1.68 [26]; SSR 1.28, 95% CI 0.55–2.51 [27]; OR 1.4, 95% CI 0.7–2.9 [29]). A moderate increase in breast cancer risk has been suggested but more evidence is needed to validate these findings [70,71].

The *NBN* encoded protein interacts with *MRE11A* and *RAD50* encoded proteins as a large complex, which interacts with the protein produced by the *ATM* gene. These combined proteins have an important role in identifying broken strands of DNA and repairing it. Due to its essential function in the DNA repair pathway and because this gene is commercially available in gene testing panels for ovarian cancer, case-controls studies have examined if they are susceptibility genes. However, most studies have not found a higher frequency of pathogenic variants in *NBN*, *MRE11A*, or *RAD50* in cases compared to controls (*NBN*: *p* 0.61 [11] and 0.09 [35]; *MRE11A*: *p* 1.0 [35]; *RAD50*: *p* 0.63 [35]) and therefore no evidence of association with risk was observed in any of these 3 genes.

### 4.4. CHECK2

Germline pathogenic variants in *CHECK2* (a cell cycle checkpoint regulator) are associated with an increased risk of breast cancer [72] and *CHECK2* was proposed to be a candidate gene for ovarian cancer risk on that basis. A prospective study had shown that in estrogen receptor–positive breast cancer, CHEK2*1100delC heterozygosity was associated with increased risk of early death, breast cancer–specific death, and risk of a second breast cancer [72]. However, numerous retrospective case-control studies did not find an association between pathogenic variants in *CHECK2* and an increased risk of ovarian cancer [26,27,35].

### 4.5. Other Genes

*TP53* was suggested as a potential candidate susceptibility gene for ovarian cancer from a case only study [34] and from a whole-exome sequencing analysis where protein truncating variants were identified at a greater frequency in ovarian cancer cases compared to publicly available controls (OR 18.5; 95% CI, 2.6–808.1) [34,57]. This gene has not been validated in other targeted sequencing and WES studies [26,35].

Other candidate genes selected for targeted sequencing validation in cases and controls have shown weak evidence of association with ovarian cancer risk for protein truncating variants in *POLK*, *SLX4* (also known as *FANCP*), and *FBXO10*, but further studies are required to confirm this [54]. Several Fanconi Anaemia genes (*FANCA*, *FANCB*, *FANCC*, *FANCD2*, *FANCE*, *FANCG*, *FANCI*, and *FANCL*) have been tested in these case-control studies but a risk association has not been detected [54]. A large number of other genes have been examined and not been shown to be ovarian cancer risk genes [48,57,66].

## 5. Discussion

We have presented a comprehensive review of the contribution of rare genetic variants to ovarian cancer, with a focus on the relationship between genetic variation and ovarian cancer histotypes, using data mostly only presented as supplementary in the original papers.

For *BRCA1/2*, deleterious germline variants are more common in HGS (8% for *BRCA1* and 6% for *BRCA2*), but there is a significant frequency of variants in END (3% for both genes), CCC (3% for *BRCA1* and 1% for *BRCA2*), and LGS (3% for *BRCA1* and 2% for *BRCA2*) patients. Consequently, the current guidelines recommend *BRCA1/2* testing in all non-mucinous ovarian cancer cases. For the MMR genes, the frequency of deleterious germline variants is higher in END and CCC patients, with variants in *MSH6* being more common than variants in *MLH1*, *MSH2*, and *PMS2* (greater than 1% in *MSH6* and approximately or less than 0.5% in *MLH1*/*MSH2/PMS2*). For *BRIP1*, the frequency of protein truncating variants seems to be similar for HGS and END (approximately 1.2%), but slightly lower for LGS cases (0.8%). For *RAD51C*, a slightly higher frequency of variants in the HGS cases is observed (0.5%), and frequencies range from 0.2 to 0.4% in END, CCC, and LGS. No variants in MUC were found. In contrast, a higher frequency of protein truncating variants in END cases is seen for *RAD51D* (0.9%), compared to the HGS cases (0.5%). No variants were detected in CCC, LGS, or MUC. A higher frequency of protein truncating variants in CCC is seen in *PALB2*, compared to 0.4% in HGS and 0.2% in LGS. However, larger numbers of the non-HGS cases are needed to confirm these frequencies especially in the moderate risk genes that have been recently confirmed as susceptibility genes for ovarian cancer.

The role of individual missense variants in the known ovarian cancer genes is not yet known. Work by the ENIGMA consortium is underway to identify deleterious missense variants in *BRCA1* and *BRCA2*, and ongoing efforts will be required for other genes [58].

The frequency of large genomic alternations (insertions, deletions, and rearrangements) have been examined in *BRCA1/2* and the MMR genes [17,73]. These types of changes make up 8–40% of *BRCA1* mutations, depending on the population [17]. We do not yet know how frequent they are in the *BRIP1*, *RAD51C*, *RAD51D*, and *PALB2* genes, and this could affect prevalence estimates. These types of changes cannot be detected with the methods currently used for targeted sequencing in clinical panel testing.

As outcomes in ovarian cancer are linked to its stage at diagnosis, the ability to identify women at risk earlier or before diagnosis has important clinical implications. The use of multigene panel testing has allowed women to be tested for multiple genetic variants associated with increased cancer risk, enabling personalised risk estimates to be developed [74,75]. While screening for ovarian cancer has not been shown to reduce mortality, RRSO can be offered to women at a sufficiently high risk of developing ovarian cancer [4,5].

There has been some debate as to what level of lifetime cancer risk justifies prophylactic surgical intervention. Traditionally, a threshold of greater than 10% lifetime ovarian cancer risk was used, allowing for RRSO in women carrying *BRCA1*, *BRCA2*, or mismatch repair gene mutations, which confer lifetime ovarian cancer risk well in excess of 10% [76]. The more recent discovery of moderate penetrance genes has prompted formal studies into risk thresholds for invention, and recent analyses have suggested that offering RRSO to women with a lifetime risk as low as 4–5% can be cost-effective [50]. On that basis, women carrying variants in *BRIP1*, *RAD51C*, and *RAD51D* may also be offered RRSO [77,78].

The best approach to managing patients with genetic variants that do not sufficiently increase ovarian cancer risk to justify RRSO but increase risk to higher than that of the general population is not yet known. While it is plausible that targeting these women for screening may allow for detection of cancers at an earlier stage, this has not been studied. Studies investigating the impact of ovarian cancer screening using ultrasonographic and biochemical methods in both the general population, and high-risk groups (greater than 10% risk on the basis of family history or presence of genetic variants) have been associated with stage-shift, but have not been shown to significantly reduce mortality [5,79].

**Figure 1:**
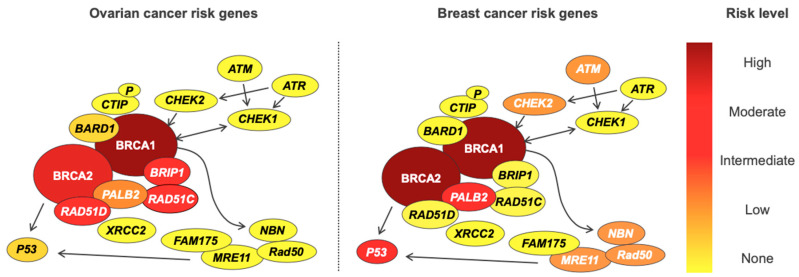
Susceptibility genes present in the double-strand DNA break repair pathway for ovarian and breast cancer and its different correspondent risks levels in each disease.

Genetic testing is fraught with ethical, legal, and psychosocial implications for patients, and the rate of advancement in our understanding of cancer genetics often outstrips our ability to use information in the clinic [80,81]. It is important to note that genetic susceptibility to EOC cancer does not exist in isolation, and variants conferring increased ovarian cancer risk also often increase the risk of developing other cancers—most notably breast cancer in *BRCA1* and *BRCA2*, and colon cancer in Lynch syndrome. Furthermore, breast and ovarian cancer genes are often combined into a single panel test, potentially uncovering variants in genes for which pathogenicity is disputed. As an example, *PALB2* is associated with a lifetime breast cancer risk of 53%, but also lifetime risks of 5% and 2–3% for ovarian and pancreatic malignancies, respectively [12]. While a 53% lifetime breast cancer risk is clearly clinically actionable, the risk of ovarian and pancreatic cancers associated with *PALB2* exists in an area of clinical uncertainty. The opposite is seen for deleterious variants in *BRIP1*, with an increased risk of ovarian cancer but no increase in breast cancer risk. For these reasons, all women undergoing genetic testing for familial susceptibility to ovarian cancer should receive appropriate pre- and post-test counselling, and follow-up with relevant clinical services. Genetic information should be applied cautiously in the clinic, after careful evaluation of the available literature, as some genes included on so called “ovarian cancer” panel tests have now been shown not to increase ovarian cancer risk [82,83] (Table 6). The risks of ovarian and breast cancer for genes in the double-strand DNA break repair pathway are shown in Figure 1.

Current testing guidelines do not recommend population-based genetic testing for ovarian cancer, however population-based testing approaches may be effective in groups with a small number of common founder mutations such as the three *BRCA1/2* variants in Ashkenazi Jews [86,87]. With the decreasing costs of sequencing methods, these approaches may also become cost effective in other populations. Population-based genetic testing has many issues, including cultural and psychosocial, that need to be investigated [87]. The majority of the genetic studies in ovarian cancer have been performed in white populations, and work on other ethnic groups is ongoing [88].

Identifying ovarian cancer genes has translated to novel therapeutic options for patients. PARP inhibitors, which target double-stranded DNA repair in cells with deficient homologous recombination, including cells with dysfunctional BRCA DNA repair pathways, have been shown to improve progression free survival (PFS) in serous and endometrioid ovarian cancers [89,90,91,92]. The impact of PARP inhibitors on PFS is most dramatic in patients with mutations in *BRCA1* and *BRCA2*, but can also benefit women without BRCA mutations if they are found to have deficiencies in homologous recombination, such as variants in the DNA repair genes discussed above [92]. However, improvements in PFS do not necessarily translate to improved overall survival, and the impact of PARP inhibitors on the overall survival endpoint has not yet been reported [92].

To date, ovarian cancer has largely been treated as a single entity. However, as outlined above, the five main histotypes of epithelial ovarian cancer appear to be characterised by distinct genetic mutations. The tumours also have different molecular profiles, and patients have different treatment responses [93]. Therefore, it has become increasingly clear that ovarian cancer represents not just a single disease but encompasses a number of distinct cancers. Most of our understanding of ovarian cancer genes relates to high-grade serous tumours, as they represent the majority of ovarian cancers. Identifying genetic variants contributing to non-serous ovarian cancers has posed an ongoing challenge for researchers, as finding rare variants for rare cancers requires studies with sample sizes that are not yet feasible.

Despite significant advancements in our understanding of the genetic epidemiology of ovarian cancer, the known ovarian cancer risk variants explain less than half of the excess familial risk for ovarian cancer [8]. Efforts to identify novel genetic variants associated with ovarian cancer are ongoing. The discovery of new genetic variants should be accompanied by efforts to accurately quantify the exact magnitude of increased risk associated with that variant. Case-control studies, the most commonly used study design in gene discovery, are only able to generate relative risks, from which absolute risks are extrapolated.

Prospective population-based studies, which have been used to provide gold-standard estimates of lifetime cancer risk in *BRCA1*, *BRCA2*, and the mismatch repair genes, would provide more accurate risk estimates for moderate penetrance ovarian cancer genes, but are costly, and require long-term investment. Segregation analysis from large international consortia has been used to calculate ovarian cancer risks in *RAD51C*, *RAD51D*, and *PALB2*, and could be applied to other moderate risk ovarian cancer genes [10,12].

Genetic association studies, by their design, are unable to establish a causal relationship between germline variant status and cancer phenotype. The discovery of novel ovarian cancer risk genes should be followed-up by further efforts to characterise the functional mechanisms by which they contribute to cancer development and identify potential therapeutic targets.

## 6. Conclusions

Ovarian cancer is the most lethal gynaecological malignancy, and an improved understanding of the contribution of rare genetic variants to the development of ovarian cancer allows for better clinical management of at-risk women. Based on the best available evidence, variants in *BRCA1*, *BRCA2*, *BRIP1*, *RAD51C*, *RAD51D*, and the mismatch repair genes confer ovarian cancer risks that warrant the consideration of risk-reducing surgery. The best approach for managing women with deleterious variants in ovarian cancer genes that do not warrant prophylactic surgery requires further investigation.

## Figures and Tables

**Table 1:** Frequency of germline pathogenic variants in *BRCA1/2* in epithelial ovarian cancer (EOC) histotypes, in cases from population studies.

Gene	Study	Cases Carriers/Total (Frequency)							Controls Carriers/Total (Frequency)		OR (95% CI)	Cumulative Risk (95% CI) ^a^
		HGS	END	CCC	LGS	MUC	Mixed/ Unk ^b^	All EOC	Matched	Publicly Available		
*BRCA1*	Walsh et al., 2011 [34]	31/242 **	2/23	1/17	**	-	6/78	40/360	-	-	NC	NC
		(12.8%)	(8.7%)	(5.9%)			(7.7%)	(11.1%)				
	Alsop et al., 2012 ^†^ [32]	74/709 **	7/119	4/63	**	-	3/110	88/1001	-	-	NC	NC
		(10.4%)	(5.9%)	(6.3%)			(2.7%)	(8.79%)				
	Song et al., 2014 ^ [33]	58/1105 *	3/322	3/192	6/172	1/157	13/274	84/2222	1/1528	-	60 ^#^ (10–2100)	61% (15–99%)
		(5.2%)	(0.9%)	(1.6%)	(3.5%)	(0.6%)	(4.7%)	(3.78%)	(0.07%)			
	Norquist et al., 2016 [35]	155/1498	4/77	4/58	3/70	0/16	16/196	142/1915	-	114/36276	48.9 (24–100)	NC
		(10.3%)	(5.2%)	(6.9%)	(4.3%)	(0%)	(8.2%)	(7.41%)		(0.31%)		
	**Total**	**278/3554**	**16/541**	**12/330**	**9/242**	**1/173**	**38/658**	**354/5498**	**1/1528**	**-**	**-**	**-**
		**(7.82%)**	**(2.95%)**	**(3.63%)**	**(3.72%)**	**(0.58%)**	**(5.77%)**	**(6.43%)**	**(0.07%)**			
*BRCA2*	Walsh et al., 2011 [34]	18/242 **	0/23	0/17	**	-	5/78	23/360	-	-	NC	NC
		(7.4%)	(0%)	(0%)			(6.4%)	(6.38%)				
	Alsop et al., 2012 ^†^ [32]	44/709 **	3/119	0/63	**	-	6/110	53/1001	-	-	NC	NC
		(6.2%)	(2.5%)	(0%)			(5.4%)	(5.29%)				
	Song et al., 2014 ^ [33]	64/1105 *	10/322	3/192	4/172	1/157	12/274	94/2222	4/1528	-	17 ^#^ (6.3–63)	24% (10–62%)
		(5.8%)	(3.1%)	(1.6%)	(2.3%)	(0.6%)	(4.4%)	(4.23%)	(0.26%)			
	Norquist et al., 2016 [35]	85/1498	3/77	0/58	1/70	0/16	9/196	98/1915	-	149/36276	14 (8.2–23.8)	NC
		(5.7%)	(3.9%)	(0%)	(1.4%)	(0%)	(4.6%)	(5.11%)		(0.41%)		
	**Total**	**211/3554**	**16/541**	**3/330**	**5/242**	**1/173**	**32/658**	**268/5498**	**4/1528**	**-**	**-**	**-**
		**(5.93%)**	**(2.95%)**	**(0.91%)**	**(2.0%)**	**(0.57%)**	**(4.86%)**	**(4.87%)**	**(0.26%)**			

-: Not available, NC: Not calculated due to no controls or not described, ** Frequency of serous combined, * Frequency of HGS and serous undifferentiated, ^#^ Odds ratio (OR) for the matched controls analysis, ^a^ Risks based on all cases combined, ^b^ Mixed/Unknown includes some other histotypes, ^†^ Mucinous ovarian cancer cases were excluded, ^ Not screened for large genomic alterations. Bold: highlight info.

**Table 2:** Frequency of germline protein truncating variants in *BRIP1*, *RAD51C*, *RAD51D*, and *PALB2* in EOC histotypes, in cases from population studies.

Gene	Study	Cases Carriers/Total (Frequency)							Controls Carriers/Total (Frequency)		Risk (95% CI)	Cumulative Risk (95% CI) ^a^
		HGS	END	CCC	LGS	MUC	Mixed/ Unk ^b^	All EOC	Matched	Publicly Available		
*BRIP1*	Walsh et al., 2011 [34]	2/242 **	1/23	0/17	**	-	1/78	4/360	-	-	NC	NC
		(0.8%)	(4.3%)	(0%)			(1.3%)	(1.1%)				
	Ramus et al., 2015 [11]	26/2535 *	0/65	0/25	4/416	0/26	0/160	30/ 3227	3/3444	-	RR: 11.2 ^#^ (3.2–34.1)	5.8% (3.6–9.1%)
		(1.0%)	(0%)	(0%)	(0.9%)	(0%)	(0%)	(0.92%)	(0.09)			
	Norquist et al., 2016 [35]	22/1498	1/77	0/58	0/70	0/16	3/196	26/1915	-	60/36276 ^c^	OR: 6.4 (3.8–10.6)	NC
		(1.5%)	(1.3%)	(0%)	(0%)	(0%)	(1.5%)	(1.36%)		(0.17%)		
	**Total**	**50/4275**	**2/165**	**0/100**	**4/486**	**0/42**	**4/434**	**60/5502**	**3/3444**	**-**	**-**	**-**
		**(1.17%)**	**(1.21%)**	**(0%)**	**(0.82%)**	**(0%)**	**(0.9%)**	**(1.1%)**	**(0.09%)**			
*RAD51C*	Song et al., 2015 ^†^ [48]	10/1806 *	1/383	1/225	1/405	0/166	1/444	14/3429	2/2772	-	OR: 5.2 ^#^ (1.1–24)	5.2% ^¶^ (1.1–22%)
		(0.6%)	(0.3%)	(0.4%)	(0.2%)	(0%)	(0.2%)	(0.40%)	(0.07%)			
	Norquist et al., 2016 ^†^ [35]	7/1498	1/77	0/58	0/70	0/16	3/196	11/1915	-	39/36276 ^c^	OR: 3.4 (1.5–7.6)	NC
		(0.5%)	(1.3%)	(0%)	(0%)	(0%)	(1.5%)	(0.57%)		(0.11%)		
	**Total**	**17/3304**	**2/460**	**1/283**	**1/475**	**0/182**	**4/640**	**25/5344**	**2/2772**	**-**	**-**	**-**
		**(0.51%)**	**(0.43%)**	**(0.35%)**	**(0.21%)**	**(0%)**	**(0.62%)**	**(0.47%)**	**(0.07%)**			
*RAD51D*	Song et al., 2015 [48]	9/1806 *	3/383	0/225	0/405	0/166	0/444	12/3429	1/2772	-	OR: 12 ^#^ (1.5–90)	12%^¶^ (1.5–60%)
		(0.5%)	(0.8%)	(0%)	(0%)	(0%)	(0%)	(0.35%)	(0.04%)			
	Norquist et al., 2016 [35]	7/1498	1/77	0/58	0/70	0/16	3/196	11/1915	-	14/36276 ^c^	OR: 10.9 (4.6–26.0)	NC
		(0.5%)	(1.3%)	(0%)	(0%)	(0%)	(1.5%)	(0.57%)		(0.04%)		
	**Total**	**16/3304**	**4/460**	**0/283**	**0/475**	**0/182**	**3/640**	**23/5344**	**1/2772**	**-**	**-**	**-**
		**(0.48%)**	**(0.87%)**	**(0%)**	**(0%)**	**(0%)**	**(0.47%)**	**(0.43%)**	**(0.04%)**			
*PALB2*	Ramus et al., 2015 [11]	6/2535 *	0/65	1/25	1/416	0/26	1/160	9/3227	3/3444	-	NC	NC
		(0.24%)	(0%)	(4%)	(0.24%)	(0%)	(0.62%)	(0.28%)	(0.09%)			
	Norquist et al., 2016 [35]	9/1498	0/77	1/58	0/70	0/16	2/196	12/1915	-	39/36276 ^c^	OR: 4.4 (2.1–9.1)	NC
		(0.60%)	(0%)	(1.7%)	(0%)	(0%)	(1.0%)	(0.62%)		(0.10%)		
	Song et al., 2019 [54]	18/5123	-	-	-	-	-	18/5123	6/5202	-	OR: 3.01 ^#^ (1.6–5.7)	3.2% (1.8–5.7%)
		(0.35%)						(0.35%)	(0.12%)			
	**Total ^**	**15/4033**	**0/142**	**2/83**	**1/486**	**0/42**	**3/356**	**21/5142**	**3/3444**	**-**	**-**	**-**
		**(0.37%)**	**(0%)**	**(2.4%)**	**(0.2%)**	**(0%)**	**(0.84%)**	**(0.41%)**	**(0.09%)**			

Abbreviations: RR—Relative risk; OR—odds ratio; SRR—Standardized risk ratio; -: Not available, NC: Not calculated due to no controls or not described, ** Frequency of serous combined, * Frequency of HGS and serous undifferentiated, ^#^ OR for the matched controls analysis, ^†^ Studies included one known deleterious missense variant [55], ^a^ Risks based on all cases combined, ^b^ Mixed/Unknown includes some other histotypes, ^c^ ExAC NFE-nonTCGA, ^¶^ Cumulative risks by age 70, ^ Total does not include Song et al., 2019 [54], study as extensive overlap with Ramus et al., 2015 [11]. Bold: highlight info.

**Table 3:** Frequency of germline pathogenic variants in *BRIP1*, *RAD51C*, *RAD51D*, and *PALB2*, in EOC cases from family history studies.

Gene	Study	Type of Study	Cases Carriers/Total (Frequency)	Controls (Carriers/Total Frequency)		Risk (95% CI)	Cumulative Risk (95% CI)
			All EOC	Matched	Publicly Available		
*BRIP1*	Lilyquist et al., 2017 ^¶^ [27]	Clinical testing lab	58/6294 (0.92%)	-	ExAC	SRR: 4.99 (3.8–6.4)	NC
	Kurian et al., 2017 ^¶^ [26]	Clinical testing lab	36/5020 (0.71%)	161/64,649 (0.24%)	-	OR: 2.62 (1.7–3.9)	NC
	Castera et al., 2018 ^¶^ [28]	Genetic counselling	21/4408 (0.48%)	-	72/36,276 ^a^ (0.20%)	OR: 3.77 (0.7–9.4)	NC
	Suszynska et al., 2020 [45]	Meta-analysis ^	200/22,494 (0.89%)	-	209/115,375 ^b^ (0.18%)	OR: 4.94 (4.0–6.0)	NC
*RAD51C*	Meindl et al., 2010 [46]	Family study	6/480 families	-	-	NC	NC
	Lilyquist et al., 2017 ^¶^ [27]	Clinical testing lab	44/6294 (0.7%)	-	ExAC	SRR: 5.12 (3.7–6.9)	NC
	Kurian et al., 2017 ^¶^ [26]	Clinical testing lab	32/5020 (0.6%)	72/64,649	-	OR: 4.98 (3.0–8.0)	NC
	Castera et al., 2018 ^¶^ [28]	Genetic counselling	23/4309 (0.5%)	-	43/36,276 ^a^ (0.12%)	OR: 14.6 (5.3–29.5)	NC
	Suszynska et al., 2019 [29]	Meta-analysis ^^	21/3791 (0.6%)	-	-	OR: 4.3 (2.5–7.5)	NC
	Yang et al., 2020 [10]	*RAD51C* families ^#^	125 families	-	-	RR: 7.55 (5.6–10.2)	11% (15–29%)
	Suszynska et al., 2020 [45]	Meta-analysis ^	149/23,802 (0.62%)	-	130/115,475 ^b^ (0.11%)	OR: 5.59 (4.4–7.0)	NC
*RAD51D*	Loveday et al., 2011 [47]	Family study	8/911 families	1/1060 (0.09%)	-	RR: 6.30 (2.8–13.8)	NC
	Lilyquist et al., 2017 ^¶^ [27]	Clinical testing lab	11/5743 (0.2%)	-	ExAC	SRR: 6.34 (3.1–11.3)	NC
	Kurian et al., 2017 ^¶^ [26]	Clinical testing lab	9/5020 (0.2%)	40/64,649 (0.06%)	-	OR: 4.78 (2.1–10.7)	NC
	Castera et al., 2018 ^¶^ [28]	Genetic counselling	9/4011 (0.2%)	-	18/36,276 ^a^ (0.05%)	OR: 11.8 (1.1–40)	NC
	Suszynska et al., 2019 [29]	Meta-analysis ^^	19/3258 (0.6%)	-	-	OR: 11.6 (5.9–23)	NC
	Yang et al., 2020 [10]	*RAD51D* families ^#^	60 families	-	-	RR: 7.6 (5.6–10.3)	13% (7–23%)
	Suszynska et al., 2020 [45]	Meta-analysis ^	94/22,787 (0.45%)	-	72/120,688 ^b^ (0.06%)	OR: 6.9 (5.1–9.4)	NC
*PALB2*	Yang et al., 2020 [12]	*PALB2* families ^#^	524 families	-	-	RR: 2.91 (1.4–6.0)	5% (2–10%)

Abbreviations: RR —Relative risk; OR—odds ratio; SRR—Standardized risk ratio; PVs—Pathogenic variants; -: Not available, NC: Not calculated, ^a^ ExAC NFE non-TCGA, ^b^ gnomAD NFE non-TCGA, ^¶^ deleterious missense changes included, ^ [45] Overlapped with 6 studies included in this review; ^^ [29] Overlapped with 1 study included in this review; ^#^ Segregation analysis. Bold: highlight info.

**Table 4:** Frequency of germline pathogenic variants in the mismatch repair (MMR) genes in EOC histotypes, in cases from population studies.

Gene	Study	Cases Carriers/Total (Frequency)							Controls Carriers/Total (Frequency)
		HGS	END	CCC	LGS	MUC	Mixed/ Unk ^a^	All EOC	Matched
*MLH1*	Song et al., 2014 [33]	0/1105 *	0/322	1/192	0/172	0/157	0/274	1/2222	2/1528
		(0%)	(0%)	(0.52%)	(0%)	(0%)	(0%)	(0.04%)	(0.13%)
	Norquist et al., 2016 [35]	0/1498	0/77	0/58	0/70	0/16	1/196 ^b^	1/1915	-
		(0%)	(0%)	(0%)	(0%)	(0%)	(0.51%)	(0.05%)	
	**Total**	**0/2603**	**0/399**	**1/250**	**0/242**	**0/173**	**1/470**	**2/4137**	**2/1528**
		**(0%)**	**(0%)**	**(0.40%)**	**(0%)**	**(0%)**	**(0.21%)**	**(0.04%)**	**(0.13%)**
*MSH2*	Song et al., 2014 [33]	1/1105 *	0/322	1/192	0/172	0/157	0/274	2/2222	0/1528
		(0.09%)	(0%)	(0.52%)	(0%)	(0%)	(0%)	(0.09%)	(0%)
	**Total**	**1/1105**	**0/322**	**1/192**	**0/172**	**0/157**	**0/274**	**2/2222**	**0/1528**
		**(0.09%)**	**(0%)**	**(0.52%)**	**(0%)**	**(0%)**	**(0%)**	**(0.09%)**	**(0%)**
*MSH6*	Walsh et al., 2011 [34]	0/242 **	2/23	0/17	**	-	0/78	2/360	-
		(0%)	(8.7%)	(0%)			(0%)	(0.55%)	
	Song et al., 2014 [33]	4/1105 *	2/322	3/192	0/172	0/157	0/274	9/2222	1/1528
		(0.36%)	(0.62%)	(1.56%)	(0%)	(0%)	(0%)	(0.40%)	(0.06%)
	Norquist et al., 2016 [35]	1/1498	2/77	0/58	0/70	0/16	0/196	3/1915	-
		(0.06%)	(2.59%)	(0%)	(0%)	(0%)	(0%)	(0.15%)	
	**Total**	**5/2845**	**6/422**	**3/267**	**0/242**	**0/173**	**0/548**	**14/4497**	**1/1528**
		**(0.17%)**	**(1.42%)**	**(1.12%)**	**(0%)**	**(0%)**	**(0%)**	**(0.31%)**	**(0.06%)**
*PMS2*	Song et al., 2014 [33]	0/1105 *	1/322	0/192	0/172	0/157	0/274	1/2222	0/1528
		(0%)	(0.31%)	(0%)	(0%)	(0.6%)	(0%)	(0.04%)	(0%)
	Norquist et al., 2016 [35]	4/1498	0/77	0/58	0/70	0/16	0/196	4/1915	-
		(0.3%)	(0%)	(0%)	(0%)	(0%)	(0%)	(0.20%)	
	**Total**	**4/2603**	**1/399**	**0/250**	**0/242**	**0/173**	**0/470**	**5/4137**	**0/1528**
		**(0.15%)**	**(0.25%)**	**(0%)**	**(0%)**	**(0%)**	**(0%)**	**(0.12%)**	**(0%)**
All 4 genes	**Total ^$^**	**10/2845**	**7/422**	**5/267**	**0/242**	**0/173**	**1/548**	**23/4497**	**3/1528**
		**(0.35%)**	**(1.65%)**	**(1.87%)**	**(0%)**	**(0%)**	**(0.18%)**	**(0.51%)**	**(0.19%)**

-: Not available, ** serous combined, * HGS and serous undifferentiated, ^a^ Mixed/Unknown includes some other histotypes, ^b^ *MLH1* variant found in carcinosarcoma, ^$^ Totals did not include overlapped studies. Bold: highlight info.

**Table 5:** Predicted deleterious missense variants in known moderate risk genes for EOC.

Genes	Study	Cases Del Missense/Total (Frequency)	Predicted Deleterious	Evidence of Risk from RAML
*BRIP1*	Ramus et al., 2015 [11]	35/3227 ^a^ (1.1%)	SIFT, PolyPhen-2 and Provean	Yes (All EOC, but stronger in HGS)
	Suszynska et al., 2020 [45]	2 */22494	Described in ClinVar	-
*RAD51C*	Song et al., 2015 [11]	12/3429 ^b^ (0.32%)	SIFT, PolyPhen-2 and Provean	Yes (All EOC, but stronger in HGS)
	Lilyquist et al., 2017 [27]	2 ^$^/6294	Described in ClinVar	-
	Norquist et al., 2016 [35]	1 ^#^/1915 ^c^	Functional assay [65]	-
	Suszynska et al., 2020 [45]	3 ^/22,494	Described in ClinVar	-
*RAD51D*	Song et al., 2015 [11]	16/3429 (0.46%)	SIFT, PolyPhen-2 and Provean	Yes (Only in HGS)
	Suszynska et al., 2020 [45]	1 ^¶^/22,494	Described in ClinVar	-
*PALB2*	Ramus et al., 2015 [11]	26/3227 (0.80%)	SIFT, PolyPhen-2 and Provean	No
	Song et al., 2019 [54]	40/5123 ^a^ (0.78%)	SIFT, PolyPhen-2 and Provean	No

-: Not available, ^a^ All variants reported in HGS, ^b^ One variant found in LGS – (histotype not available for the other variants), ^c^ Variant found in a serous carcinoma. *ClinVar reported variants:* * p.Gln169 = and p.Ala349Pro; ^$^ p.C135Y and p.L138F; ^#^ p.Q143R; ^ p.Cys135Tyr, p.Cys135Ser and p.Leu138Phe; ^¶^ p.Ser207Leu.

**Table 6:** Summary of the frequency of deleterious variants by EOC histotype for each homologous recombination (HR) gene and the MMR genes, and a comparison of risk and clinical management for ovarian and breast cancer patients.

Genes	Frequency (%)					Risk Est. ^		Risk Level		Clinical Management ^$^	
	HGS	END	CCC	LGS	MUC	EOC	BC	EOC	BC	EOC	BC
*BRCA1*	7.8	2.9	3.6	3.7	<1	60	72% *	Very high	Very high	RRSO age 35 to 45 PARPi	RRM age 25 to 40
*BRCA2*	5.9	2.9	<1	2	<1	17	69% *	High	Very high		
*MMR*	<1	1.6	1.9	0	0	2.3	-	Mod	None	RRSO with hysterectomy for LS	No increased risk
*BRIP1*	1.2	1.2	0	<1	0	11.2	-	Mod	None	RRSO age 45 to 50 no consensus	Insufficient evidence
*RAD51C*	<1	<1	<1	<1	0	5.2	1.9	Mod	None		
*RAD51D*	<1	<1	0	0	0	12	1.8	Mod	None		
*PALB2*	<1	0	2.4	<1	0	3.0	7.2	Low	Mod	Insufficient evidence	Annual mammography/breast MRI age 30 no consensus
*TP53*	Insufficient data					Insuf	Insuf	Low	Mod	Insufficient evidence	Insufficient evidence
*CHEK2*	No increased risk					-	3.0	None	Low	No increased risk	Annual mammography/breast MRI age 40 no consensus
*ATM*	No increased risk					-	2.8	None	Low		
*NBN*	No increased risk					-	2.7	None	Low		
*RAD50*	No increased risk					-	Insuf	None	Low	No increased risk	Insufficient evidence
*MRE11A*	No increased risk					-	Insuf	None	Low		

Abbreviations: BC—breast cancer; RRM—Risk-reducing mastectomy; LS—Lynch Syndrome; MRI—Magnetic Resonance Imaging; Insuf—Insufficient. ^ From Table 1, Table 2 and Table 4. EOC *BRCA1/2* from ref [33], MMR from ref [33], *BRIP1* from ref [11], *RAD51C/D* from ref [48], *PALB2* from ref [54]. BC *BRCA1/2* from ref [9], *RAD51C/D* from ref [10], *PALB2* from ref [12], *CHEK2/ATM/NBN* from ref [84]. ^$^ *BRCA1/2* from refs [39,85], MMR from ref [13], *BRIP1/RAD51C/D* from ref [49], *PALB2/CHEK2/ATM/NBN* from refs [77,84]. * Cumulative risk data by the age of 80.
